# Investigating the effects of perturbations to *pgi* and *eno* gene expression on central carbon metabolism in *Escherichia coli* using ^13^ C metabolic flux analysis

**DOI:** 10.1186/1475-2859-11-87

**Published:** 2012-06-21

**Authors:** Yuki Usui, Takashi Hirasawa, Chikara Furusawa, Tomokazu Shirai, Natsuko Yamamoto, Hirotada Mori, Hiroshi Shimizu

**Affiliations:** 1 Department of Bioinformatic Engineering, Graduate School of Information Science and Technology, Osaka University, 1-5 Yamadaoka, Suita, Osaka, 565-0871, Japan; 2 Quantitative Biology Center, RIKEN, 6-2-3 Furuedai, Suita, Osaka, 565-0874, Japan; 3 Catalysis Science Laboratory, Mitsui Chemicals, Inc, 1900 Togo, Mobara, Chiba, 297-8666, Japan; 4 Graduate School of Biological Sciences, Nara Institute of Science and Technology, 8916-5 Takayama, Ikoma, Nara, 630-0192, Japan; 5 Present address: RIKEN Biomass Engineering Program, 1-7-22 Suehiro-cho, Tsurumi-ku, Yokohama, Kanagawa, 230-0045, Japan; 6 Present address: Institute for Research in cities, Kyoto University, Yoshidahonmachi, Sakyo-ku, Kyoto, 606-8501, Japan

**Keywords:** *Escherichia coli*, Gene expression perturbations, ^13^ C Metabolic flux analysis, Metabolic sensitivity analysis, *pgi*, *eno*

## Abstract

**Background:**

It has long been recognized that analyzing the behaviour of the complex intracellular biological networks is important for breeding industrially useful microorganisms. However, because of the complexity of these biological networks, it is currently not possible to obtain all the desired microorganisms. In this study, we constructed a system for analyzing the effect of gene expression perturbations on the behavior of biological networks in *Escherichia coli*. Specifically, we utilized ^13^ C metabolic flux analysis (^13^ C-MFA) to analyze the effect of perturbations to the expression levels of *pgi* and *eno* genes encoding phosphoglucose isomerase and enolase, respectively on metabolic fluxes.

**Results:**

We constructed gene expression-controllable *E. coli* strains using a single-copy mini F plasmid. Using the *pgi* expression-controllable strain, we found that the specific growth rate correlated with the *pgi* expression level. ^13^ C-MFA of this strain revealed that the fluxes for the pentose phosphate pathway and Entner-Doudoroff pathway decreased, as the *pgi* expression lelvel increased. In addition, the glyoxylate shunt became active when the *pgi* expression level was almost zero. Moreover, the flux for the glyoxylate shunt increased when the *pgi* expression level decreased, but was significantly reduced in the *pgi*-knockout cells. Comparatively, *eno* expression could not be decreased compared to the parent strain, but we found that increased *eno* expression resulted in a decreased specific growth rate. ^13^ C-MFA revealed that the metabolic flux distribution was not altered by an increased *eno* expression level, but the overall metabolic activity of the central metabolism decreased. Furthermore, to evaluate the impact of perturbed expression of *pgi* and *eno* genes on changes in metabolic fluxes in *E. coli* quantitatively, metabolic sensitivity analysis was performed. As a result, the perturbed expression of *pgi* gene had a great impact to the metabolic flux changes in the branch point between the glycolysis and pentose phosphate pathway, isocitrate dehydrogenase reaction, anaplerotic pathways and Entner-Doudoroff pathway. In contrast, the impact of perturbed *eno* expression to the flux changes in *E. coli* metabolic network was small.

**Conclusions:**

Our results indicate that the response of metabolic fluxes to perturbation to *pgi* expression was different from that to *eno* expression; perturbations to *pgi* expression affect the reaction related to the Pgi protein function, the isocitrate dehydrogenase reaction, anaplerotic reactions and Entner-Doudoroff pathway. Meanwhile, *eno* expression seems to affect the overall metabolic activity, and the impact of perturbed *eno* expression on metabolic flux change is small. Using the gene expression control system reported here, it is expected that we can analyze the response and adaptation process of complex biological networks to gene expression perturbations.

## Background

In the post-genome era, it has been recognized that analyzing the behavior of the complex intracellular biological networks consisting of the genome, mRNA, proteins and metabolites is important for understanding cellular systems [1]. Such analysis has been called “systems biology.” In particular, the metabolic engineering field is acutely aware of the importance of systems biology research [2]. To breed industrially useful microorganisms such as those able to produce useful chemicals, genetic modifications, including overexpressions and deletions, have been conducted in host cells. Unfortunately, these modifications do not always result in the desired microorganism due to the complexity of biological networks. The lack of current knowledge regarding the complexity of biological networks limits the applicability of these modifications to the design of useful microorganism. Therefore analysis of the effects of genetic modifications on the complex biological networks will benefit future work aimed at breeding industrially useful microorganisms. In particular, detailed analysis of metabolic reaction network is required to produce useful chemicals, as the biosynthesis of target chemicals is connected to the metabolic network of the host microorganism [3].

Metabolic flux analysis (MFA) has become a powerful tool for analyzing the changes in the behavior of the intracellular metabolic network [4]. MFA involving ^13^ C isotope-labeling experiments (^13^ C-MFA) is widely used to quantitatively determine the metabolic flux distribution. In these ^13^ C-MFA experiments, the cells are cultivated on ^13^ C-labeled carbon source(s), and then, the ^13^ C-labeling information of proteinogenic amino acids is measured by mass spectrometry and nuclear magnetic resonance spectroscopy [5,6].

Previous studies have utilized ^13^ C-MFA to assess the effects of perturbations to the central carbon metabolism network on intracellular metabolism. For example, ^13^ C-MFA studies utilizing an *Escherichia coli* strain lacking the *pykF* gene, which encodes pyruvate kinase converting phosphoenolpyruvate to pyruvate, demonstrated [7,8]. In the chemostat culture of the *pykF*-knockout strain, the glycolytic flux is decreased and the fluxes for anaplerotic reactions catalyzed by phosphoenolpyruvate carboxylase and malic enzyme are increased, in comparison with the parent strain. Moreover, the expression of *zwfgnd*, and *ppc* encoding glucose-6-phosphate dehydrogenase, 6-phosphogluconate dehydrogenase, and phosphoenolpyruvate carboxylase, respectively, is increased, and the expression of *glkpgi*, and *tpi* genes encoding glucokinase, phosphoglucose isomerase and triosephosphate isomerase, respectively, is decreased. Further research on the *pgi*-knockout strain demonstrated that the flux through the glyoxylate shunt is increased, and acetate secretion is decreased in comparison with the parent strain [9-11]. Recently, Ishii et al. used 24 single-gene knockout mutant strains of *E. coli* in the glycolysis and the pentose phosphate pathway to analyze their effect on central carbon metabolism by ^13^C-MFA as well as transcriptomic, proteomic, and metabolimic techniques [12]. In addition, Nicolas et al. reported the difference in metabolic flux distributions between the wild-type, *zwf* knockout, and *zwf*-overexpressing strains of *E. coli*[13].

The majority of studies analyzing the effect of gene expression perturbations on biological networks utilize gene knockout strains as described above. However, because of the dramatic magnitude of the perturbation induced by gene knockout, unexpected, and perhaps, artificial phenomena may be observed. Moreover, these knockout strains have already adapted to the culture condition(s) during their construction, and therefore, the resulting analyses may be confounded. Therefore, analyzing the cellular states with native and perturbed gene expressions is important to understand the mechanism of the transition from native cellular state to the adapted state with complete loss of the target gene. For this purpose, other experimental system than utilization of knockout strains would be highly required.

In this study, we constructed a simple experimental system for analyzing the effect of perturbed gene expression on the behavior of intracellular biological networks in *E. coli*. Briefly, the target gene for analysis was cloned onto the single-copy plasmid under an inducible promoter and operator, and then, expressed; this disrupted the target gene on the chromosome. Subsequently, the expression levels of the target gene on the single-copy plasmid can be altered by changing the concentration of its inducer, and the effect of this change on the behavior of the intracellular biological networks can be analyzed. In addition, the transition from the initial cellular state to the complete loss of function of the target gene can also be analyzed.

In the present study, we reported the construction of expression-controllable strain for the *pgi* gene of *E. coli*. Subsequently, we analyzed the effect of perturbations to *pgi* expression levels on carbon metabolism via ^13^C-MFA. The *pgi* gene encodes the glycolytic enzyme phosphoglucose isomerase, which is an important member of the glycolysis pathway and links to the oxidative pentose phosphate pathway via its catalysis of the conversion from glucose-6-phosphate to fructose-6-phoshate [14]. In addition, our method is expected to allow the analysis of essential genes. Therefore, we constructed *eno* expression-controllable strain of *E. coli*, and analyzed the effect of perturbations to *eno* expression levels on carbon metabolism by ^13^ C-MFA. The *eno* gene also encodes a glycolytic enzyme, enolase, which catalyzes the conversion from 2-phosphoglycerate to phosphoenolpyruvate, and its knockout is lethal when grown on glucose as a carbon source [15].

MFA helps us to understand the metabolic relationship between different metabolic pathways and to quantify the distribution of fluxes in the metabolic reaction networks. However, any quantitative measures of the control of the flux in metabolic networks cannot be obtained from the results of MFA only. To evaluate the sensitivity of the metabolic fluxes to the change in activities of metabolic reactions, metabolic control analysis (MCA) has been performed [4]. In MCA, perturbation experiment to target enzyme activity has been carried out and sensitivity of metabolic fluxes to change in activity of metabolic enzymes was evaluated by estimating the flux control coefficients (FCCs). In this study, metabolic sensitivity analysis (MSA) was conducted, and the sensitivity of fluxes when changing the expression of *pgi* and *eno* genes was quantitatively evaluated. As a result, sensitivities of fluxes for the branch point between the glycolysis and pentose phosphate pathway, isocitrate dehydrogenase reaction, anaplerotic pathways and Entner-Doudoroff pathway to the change in *pgi* expression levels were large. In contrast, sensitivity of fluxes in *E. coli* metabolic network was small when changing the *eno* expression levels.

## Results

### Construction of gene expression-controllable *E. coli* strains

**Figure 1:**
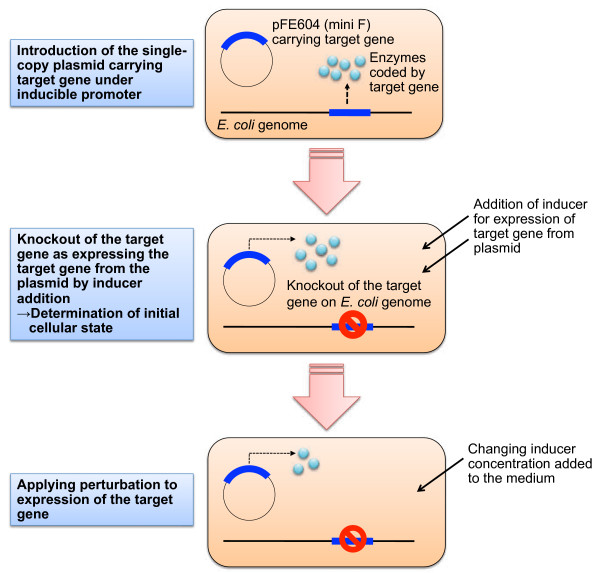
**Construction of the gene expression-controllable*E. coli*strain.** Details of the method for constructing the gene expression-controllable strain are described in the Results and Methods sections.

To investigate the effect of the changes in target gene expression levels on cellular metabolic status, gene expression-controllable strains were required. The gene expression-controllable *E. coli* strains were constructed using a single-copy mini-F plasmid pFE604 in this study (Figure 1). The target gene was cloned into pFE604 under the T5 promoter and *lac* operator, and then, the resulting plasmid was introduced into the *E. coli* BW25113 strain. Following this, the chromosomal target gene of the transformed BW25113 strain was disrupted in the presence of isopropyl-β-D-thiogalactoside (IPTG) which induces expression of the target gene on pFE604. Since the copy number of both the pFE604 plasmid and the chromosomal DNA is singular, the disrupted chromosomal gene can be substituted by the pFE604 carrying the target gene and the expression can be controlled by altered IPTG concentrations.

It should be noted that this gene expression-controllable strain cultured in the absence of IPTG is dissimilar to the knockout strain of the target gene, because the knockout strain has adapted to the culture conditions used for strain construction. The constructed strains allow the initial cellular state to be determined, and thus, we can analyze the process of change by perturbing gene expression in the initial cellular state. It is expected that the cellular state in the knockout strain is similar to that in the expression-controllable strain that has been allowed to adapt to the culture conditions in the absence of IPTG.

In this study, we constructed expression-controllable strains for *pgi*, which encodes phosphoglucose isomerase located to the branch point between glycolysis and the oxidative pentose phosphate pathway, and *eno*, which encodes enolase that functions at the later stage of glycolysis and whose knockout is lethal.

### Effect of perturbed *pgi* gene expression on cell growth in *E. coli*

We analyzed the effect of *pgi* expression levels on cellular characteristics such as cell growth and metabolic flux distribution. The preculture was prepared by incubating the *pgi* expression-controllable strain YUEC04 in modified M9 medium containing ^13^ C-labeled glucose and IPTG (20, 50 and 100 μg/mL). The main culture was made by inoculating the preculture into modified M9 medium containing ^13^ C-labeled glucose and the same concentration of IPTG as that in the preculture to achieve an optical density of culture at 660 nm (OD_660_) of 0.01. After the OD_660_ reached approximately 1, the cells were harvested for gene expression analysis using real-time reverse transcription polymerase chain reaction (RT-PCR) and ^13^ C-MFA. For comparison, YUEC00 (BW25113/pFE604) and the *pgi* knockout strain JWK3985 in the single-gene knockout strain collection, the Keio collection [16], were also cultured without IPTG addition.

**Figure 2:**
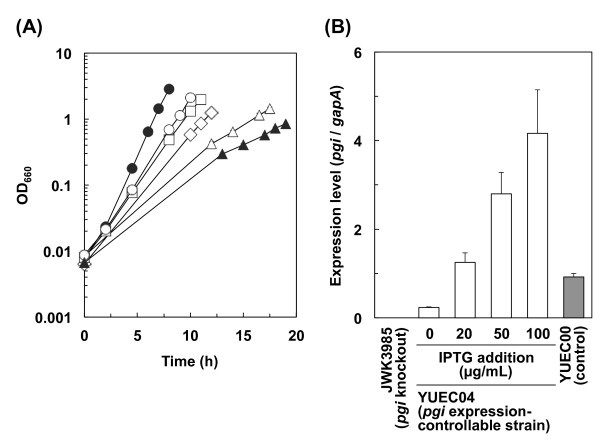
**Growth properties and*pgi*expression levels of the*pgi*expression-controllable strain.** (**A**) Growth of the *pgi* expression-controllable strain YUEC04 cultivated in modified M9 medium containing 0 μg/mL IPTG (open triangles), 20 μg/mL IPTG (open diamonds), 50 μg/mL IPTG (open squares), 100 μg/mL IPTG (open circles). In addition, the growth of the control strain YUEC00 (BW25113/pFE604; closed circles) and the *pgi*-knockout strain JWK3985 (closed triangles) are also shown. (**B**) Expression of *pgi* in the *pgi* expression-controllable strain YUEC04, control strain YUEC00, and the *pgi*-knockout strain JWK3985.

Figure 2 shows the growth properties and *pgi* expression levels in YUEC04 as well as in YUEC00 and JWK3985 in the presence and absence of various IPTG concentrations. It was noted that increased IPTG concentrations correlated with increased *pgi* expression levels and increased specific growth rate during exponential the growth phase (Figure 2 and Table 1). However, the specific growth rate of the YUEC04 strain cultured in the presence of IPTG was lower than that of the control strain YUEC00 in spite of the higher *pgi* expression levels in the former. Moreover, the growth properties of the control YUEC04 cells in the absence of IPTG were slightly different from that of the *pgi* knockout strain JWK3985. This supports the need to use the *pgi* expression-controllable YUEC04 strain for metabolic analysis of cellular states with altered *pgi* expression. Acetate was not produced in JWK3985 and YUEC04 strains; however, it was highly produced in the control YUEC00 strain (Table 1).

**Table 1:** **Specific growth, glucose consumption and acetate production rates of the** ***pgi*** **expression-controllable strain**

**Strain**	**IPTG (μg/mL)**	**Specific growth rate (1/h)**	**Specific glucose consumption rate (mmol/g dry cell/h)**	**Specific acetate production rate (mmol/g dry cell/h)**
YUEC04	0	0.23 ± 0.01	4.67 ± 0.85	0
	20	0.39 ± 0.03	5.87 ± 0.51	0
	50	0.47 ± 0.00	4.58 ± 1.94	0
	100	0.56 ± 0.05	4.91 ± 3.96	0
YUEC00	0	0.74 ± 0.01	8.77 ± 0.15	4.20 ± 0.09
JWK3985	0	0.19 ± 0.00	4.46 ± 3.07	0

Average ± standard deviation from 3 independent culture experiments is shown. Specific growth and glucose consumption rates were calculated using OD_660_ values (shown in Figure 2) and glucose concentrations in culture supernatant (data not shown) at the last three time points in each culture shown in Figure 2.

Our results indicate that perturbations to *pgi* expression affect the physiological characteristics of *E. coli* and the cellular physiological state is responsive to alteration to these perturbations.

### Effect of perturbed *pgi* gene expression on metabolic flux distribution in *E. coli*

Next, we examined the changes in metabolic flux distribution associated with altered *pgi* expression in the *pgi* expression-controllable YUEC04 strain. In addition, the metabolic flux distributions of the *pgi*-knockout strain JWK3985 and the control strain YUEC00 were also analyzed.

**Figure 3:**
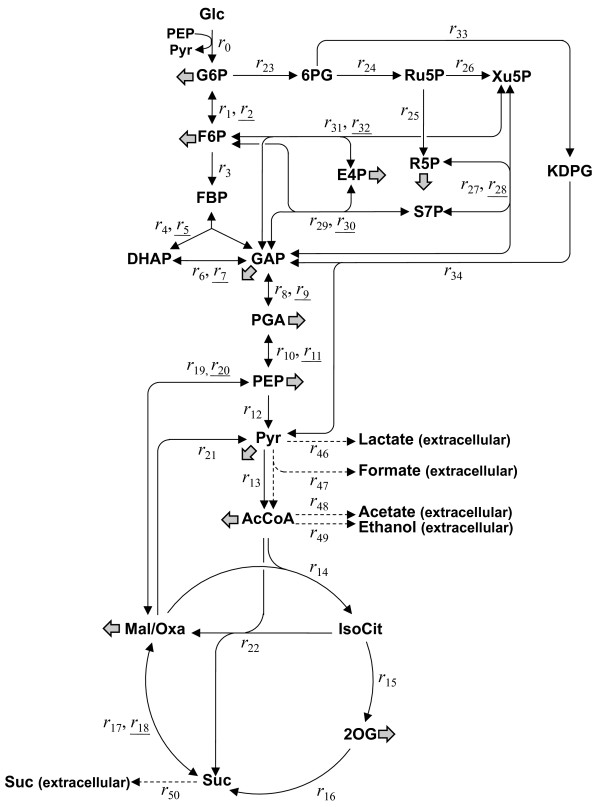
**Metabolic reaction model of*E. coli*for**^**13**^**C-MFA.** Forward and reverse (underlined) reactions are shown. Thick arrows indicate the fluxes for the synthesis of precursors for biomass production. G6P, glucose-6-phosphate; F6P, fructose-6-phosphate; FBP, fructose-1,6-bisphosphate; GAP, glyceraldehyde-3-phosphate; DHAP, dyhydroxyacetonephosphate; PGA, 3-phosphoglycerate; PEP, phosphoenolpyruvate; Pyr, pyruvate; AcCoA, acetyl-CoA; IsoCit, isocitrate; 2OG, 2-oxoglutarate; Suc, succinate; Mal, malate; Oxa, oxaloacetate; 6PG, 6-phosphogluconate; Ru5P, ribulose-5-phosphate; R5P, ribose-5-phosphate; S7P, sedohetulose-7-phosphate; X5P, xylulose-5-phosphate; E4P, erythrose-4-phosphate; KDPG, 2-keto-3-deoxyphosphogluconate.

The metabolic reaction model, including glycolysis, the pentose phosphate pathway, the tricarboxylic acid (TCA) cycle, anaplerotic reactions, and the Entner-Doudoroff pathway, was constructed (Figure 3). Although net fluxes for the inactive reactions would initially be set to zero, the flux for phosphoglucose isomerase (*r*_1_–*r*_2_) was not be set to zero in this study.

**Figure 4:**
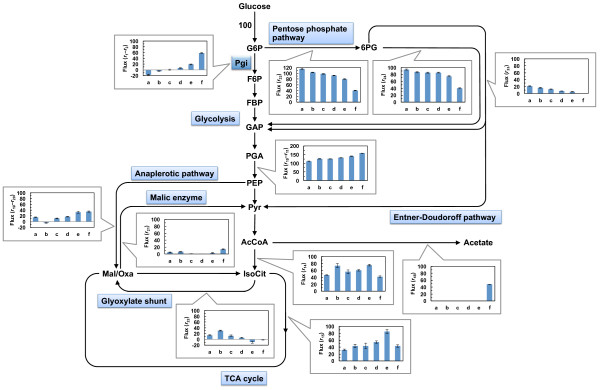
**Effect of perturbed*pgi*gene expression on metabolic flux in*E. coli*.** All fluxes were normalized to the specific glucose uptake rate of 100 to allow comparison of the flux distributions at different glucose uptake rates. Metabolic flux distributions of the *pgi*-knockout strain JWK3985 (**a**), *pgi* expression-controllable strain YUEC04 in the presence of 0 μg/mL IPTG (**b**), 20 μg/mL IPTG (**c**), 50 μg/mL IPTG (**d**), and 100 μg/mL IPTG (**e**) and the control strain YUEC00 (**f**) are shown. As for the reversible reactions, net fluxes are shown. G6P, glucose-6-phosphate; F6P, fructose-6-phosphate; FBP, fructose-1,6-bisphosphate; GAP, glyceraldehyde-3-phosphate; PGA, 3-phosphoglycerate; PEP, phosphoenolpyruvate; Pyr, pyruvate; AcCoA, acetyl-CoA; IsoCit, isocitrate; Mal, malate; Oxa, oxaloacetate; 6PG, 6-phosphogluconate.

As shown in Figure 4, and Additional file 1 and Additional file 2, the flux for phosphoglucose isomerase increased and the flux for glucose-6-phosphate dehydrogenase (*r*_23_) decreased with increasing IPTG concentrations (i.e., increasing *pgi* expression levels). Moreover, the flux for 6-phosphogluconate dehydratase (*r*_33_) in the Entner-Doudoroff pathway also decreased with increasing *pgi* expression levels. This is similar to previous reports, which have shown that the Entner-Doudoroff pathway is activated in the *pgi*-knockout strain [9-11].

The flux toward the TCA cycle from glycolysis (*r*_14_ and *r*_15_) in the YUEC04 strain was higher than that in the *pgi*-knockout and the control strains. The net flux for phosphoenolpyruvate carboxylase and phosphoenolpyruvate carboxykinase (*r*_19_–*r*_20_) in the YUEC04 strain increased concomitantly with increased *pgi* expression levels. Moreover, the flux toward the glyoxylate shunt (*r*_22_) decreased in response to increasing *pgi* expression levels, but it was higher in the *pgi* expression-controllable strain cultured in the absence of IPTG than that in the *pgi*-knockout strain. These results suggest that the flux toward the glyoxylate shunt increases concomitantly with decreasing the *pgi* expression levels, but is also significantly decreased in the complete absence of *pgi* expression. The glyoxylate shunt was less active as shown in the *pgi* expression-controllable strain cultured in the presence of 100 μg/mL IPTG. As shown in Additional file 3, the expression of *aceBA* gene was increased as decreasing the *pgi* expression level. These results suggest increased flux toward the glyoxylate shunt due to the increased *aceBA* expression levels correlated with decreased *pgi* expression levels.

It can be concluded from our results that the changes in the levels of metabolic flux induced by perturbed gene expression can be analyzed by our gene expression-controllable strain.

### Effect of perturbed *eno* gene expression on cell growth and metabolic flux distribution in *E. coli*

Given the success with our *pgi* expression-controllable strain, we decided to utilize the method to analyze the effect of perturbations to the expression of essential genes. To this end, we constructed an expression-controllable strain for the *eno* gene, which encodes enolase an essential *E. coli* enzyme responsible for catalyzing the conversion from 3-phosphoglycerate to phosphoenolpyruvate.

**Figure 5:**
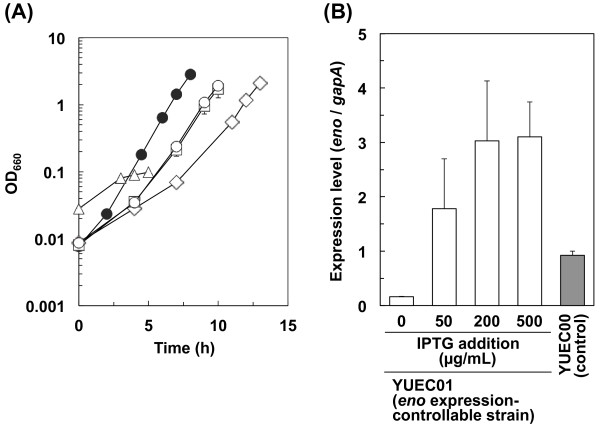
**Growth properties and*eno*expression levels of the*eno*expression-controllable strain.** (**A**) Growth of *eno* expression-controllable strain YUEC01 cultivated in modified M9 mediums containing 0 μg/mL IPTG (open triangles), 50 μg/mL IPTG (open diamonds), 200 μg/mL IPTG (open squares), and 500 μg/mL IPTG (open circles). In addition, the growth of the control strain YUEC00 (BW25113/pFE604; closed circles) is also shown. (**B**) Expression of the *eno* gene in the *eno* expression-controllable strain YUEC01, and the control strain YUEC00.

Similar to the conditions utilized for the analysis of the *pgi* expression-controllable strain, the *eno* expression-controllable strain YUEC01 was cultured in modified M9 medium containing ^13^ C-labeled glucose and 50, 200 or 500 μg/mL IPTG. In the presence of IPTG, the YUEC01 strain grew and the *eno* expression from the pFE604-*eno* plasmid was higher than that from chromosomal DNA in the YUEC00 (BW25113/pFE604) control strain (Figure 5). Moreover, the specific growth rate of the YUEC01 strain in the presence of IPTG was lower than that of the YUEC00 control strain, and the growth lag time of the YUEC01 in the presence of IPTG was longer than that of the YUEC00 control strain (Figure 5 and Table 2). These results suggest that the increased expression of the *eno* gene might be toxic to the *E. coli* cells. We could not determine the IPTG concentration that achieved lower *eno* expression levels than the control strain (data not shown).

**Table 2:** Specific growth, glucose consumption and acetate production rates of the eno expression-controllable strain

**Strain**	**IPTG (μg/mL)**	**Specific growth rate (1/h)**	**Specific glucose consumption rate (mmol/g dry cell/h)**	**Specific acetate production rate (mmol/g dry cell/h)**
YUEC01	0	Not determined	Not determined	Not determined
	50	0.67 ± 0.01	8.34 ± 0.45	4.14 ± 0.07
	200	0.62 ± 0.02	7.54 ± 0.68	4.21 ± 0.76
	500	0.58 ± 0.01	7.63 ± 0.66	3.81 ± 0.42
YUEC00	0	0.74 ± 0.01	8.77 ± 0.15	4.20 ± 0.09

Average ± standard deviation from 3 independent culture experiments is shown. Specific growth and glucose consumption rates were calculated using OD_660_ values (shown in Figure 5) and glucose concentrations in culture supernatant (data not shown) at the last three time points in each culture shown in Figure 5.

**Figure 6:**
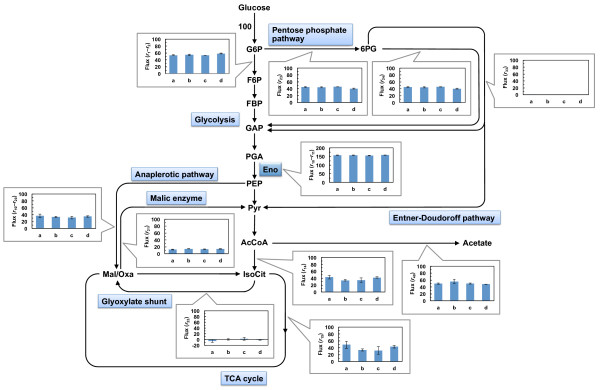
**Effect of perturbed*eno*gene expression on metabolic flux in*E. coli*.** All fluxes were normalized to the specific glucose uptake rate of 100 to allow comparison of the flux distributions at different glucose uptake rates. Metabolic flux distributions of the *eno* expression-controllable strain YUEC01 in the presence of 50 μg/mL IPTG (**a**), 200 μg/mL IPTG (**b**), and 500 μg/mL IPTG (**c**), and the control strain YUEC00 (**d**) are shown. As for the reversible reactions, net fluxes are shown. G6P, glucose-6-phosphate; F6P, fructose-6-phosphate; FBP, fructose-1,6-bisphosphate; GAP, glyceraldehyde-3-phosphate; PGA, 3-phosphoglycerate; PEP, phosphoenolpyruvate; Pyr, pyruvate; AcCoA, acetyl-CoA; IsoCit, isocitrate; Mal, malate; Oxa, oxaloacetate; 6PG, 6-phosphogluconate.

Next, the metabolic flux distribution of YUEC01 cultured in the presence of IPTG was determined. As shown in Figures 6, and Additional file 1 and Additional file 4, the metabolic flux distribution of YUEC01 cultured in the presence of IPTG was similar to that of the YUEC00 control strain. However, the specific glucose consumption rate in the presence of 50 μg/mL IPTG was higher than that in the presence of 500 and 200 μg/mL IPTG (Table 2), suggesting that the high *eno* expression level decreases the reaction rates (or activity) of overall central metabolism.

### Evaluation of the impact of perturbed expression of *pgi* and *eno* genes on metabolic fluxes by metabolic sensitivity analysis

We evaluated the impact (or sensitivity) of perturbed expression of *pgi* and *eno* genes on metabolic fluxes by MSA in which the experimental flux sensitivity coefficients (e-FSCs) were estimated (Tables 3 and 4). In this study, the sensitivity of metabolic fluxes for the reactions shown in Figures 4 and 6 to the changes in the *pgi* or *eno* expression levels were evaluated.

**Table 3:** **e-FSCs against perturbed** ***pgi*** **gene expression**

***J***_***k***_**for**$C_{\mathrm{pgi}}^{J_{k}}$^**a**^	**IPTG concentration in culture of *pgi*** **expression-controllable strain (μg/ml)**^**b**^			
	**0 (0.23)**	**20 (1.25)**	**50 (2.80)**	**100 (4.16)**
J_1_–J_2_	4.00	3.98	3.60	2.59
J_10_–J_11_	0.08	0.03	0.01	0.01
J_14_	0.00	0.06	0.14	0.15
J_15_	0.00	0.04	0.79	1.78
J_19_–J_20_	0.00	0.30	1.53	1.92
J_22_	–0.01	–0.39	–4.77	–8.57
J_23_	0.00	–0.06	–0.27	–0.55
J_24_	0.01	0.00	0.00	0.00
J_33_	–0.02	–0.28	–0.31	–0.21

^a^e-FSC was estimated from metabolic flux distributions and *pgi* expression levels of the *pgi* expression-controllable-strain YUEC04 and *pgi* knockout strain JWK3985.

^b^Value in parentheses represents the *pgi* expression level shown in Figure 2.

**Table 4:** **e-FSCs against perturbed** ***eno*** **gene expression**

***J***_***k***_**for**$C_{\mathrm{eno}}^{J_{k}}$^**a**^	**Control strain (0.92)**^**b**^	**IPTG concentration in culture of** ***eno*** **expression-controllable strain (μg/ml)**^**b**^		
		**50 (1.78)**	**200 (3.03)**	**500 (3.10)**
*J*–*J*	0.05	0.01	0.00	0.00
*J*–*J*	0.04	0.01	0.00	0.00
*J*	0.08	0.02	0.01	0.01
*J*	0.18	0.05	0.02	0.02
*J*–*J*	0.12	0.03	0.01	0.01
*J*	2.00	1.98	1.96	1.95
*J*	0.01	0.00	0.00	0.00
*J*	0.00	0.00	0.00	0.00
*J*	0.47	0.15	0.06	0.05

^a^e-FSC was estimated from metabolic flux distributions and *eno* expression levels of the *eno* expression controllable-strain YUEC01 and its control strain YUEC00.

^b^Value in parentheses represents the *eno* expression level shown in Figure 5.

As expected, change in *pgi* expression had a great impact on the metabolic fluxes for the branch point between glycolysis and the oxidative pentose phosphate pathway, i.e. phosphoglucose isomerase (*r*_1_–*r*_2_) encoded by *pgi* and glucose-6-phosphate dehydrogenase (*r*_23_) (Table 3). Moreover, it also had an impact on the fluxes for isocitrate dehydrogenase (*r*_15_), anaplerotic reactions (*r*_19_–*r*_20_ and *r*_22_) and Entner-Doudoroff pathway (*r*_33_). Particularly, the e-FSCs for *r*_22_, *r*_23_ and *r*_33_ were negative, indicating that the perturbed *pgi* expression has a negative impact to these metabolic reactions. In addition, low *pgi* expression level exhibited an impact to the metabolic flux change for phosphoglucose isomerase (*r*_1_–*r*_2_). In contrast, high *pgi* expression level had an impact to metabolic flux change for isocitrate dehydrogenase (*r*_15_), anaplerotic reactions (*r*_19_–*r*_20_ and *r*_22_), glucose-6-phosphate dehydrogenase (*r*_23_) and Entner-Doudoroff pathway (*r*_33_). Notably, the positive impact to flux change for isocitrate dehydrogenase (*r*_15_) and negative impact to flux change for glyoxylate shunt (*r*_22_) were large.

On the other hand, the impact of perturbation to *eno* gene expression on metabolic fluxes was small except for the glyoxylate shunt (*r*_22_), but this phenomenon might be caused by the low flux of this reaction.

## Discussion

Analysis of the biological networks consisting of the genome, mRNAs, proteins and metabolites is of particular importance to the metabolic engineering field [2]. These analyses will hopefully help us obtain the desired microorganism via genetic modifications such as gene overexpression and deletion. In this study, we analyzed the effect of gene expression perturbations on cellular metabolism in *E. coli* by using ^13^ C-MFA. Generally, previous studies designed to analyze the effect of perturbed gene expression have utilized gene knockout strains. However, since the magnitude of the perturbation by gene knockout is high, unexpected phenomena might occur. Moreover, such knockout strains have already adapted to the culture conditions during their construction, and therefore, analysis after this adaptation process has occurred will lead to confounding results. To avoid these complications, we constructed a simple experimental system for changing gene expression levels artificially via a single-copy mini-F plasmid carrying an IPTG-inducible promoter. In our system, knockout of the chromosomal target gene is performed concomitantly with expressing the target gene on the mini-F plasmid carrying the target gene by IPTG addition. This allows the initial cellular state, prior to altered target gene expression, to be determined. Moreover, a range of intracellular expression levels of target gene can be created by changing the IPTG concentration. For the same objective, exchange of the promoter of the target gene on the genome DNA to the inducible promoter, such as *tetA* promoter, might be able to be carried out alternatively [17]. This strategy is thought to be useful, but it might be necessary to consider the polar effect of expression of the genes located at the downstream of target gene. In our experimental system, the effect of the perturbation to only target gene expression can be analyzed by integrating inducible expression of target gene on a single-copy plasmid with the in-frame gene knockout, which is the same strategy as the Keio collection [16]. However, further improvement of our experimental system would be considered.

We utilized our gene expression control system to analyze the effect of perturbed *pgi* and *eno* expression on cellular metabolism in *E. coli*. The *pgi* gene encodes phosphoglucose isomerase converting glucose-6-phosphate to fructose-6-phosphate which functions at the branch point between the glycolytic and oxidative pentose phosphate pathways. Previously, ^13^ C-MFA of the *pgi*-knockout strain was performed in glucose-limited chemostat and batch cultures, revealing that the pentose phosphate pathway is a major route for glucose catabolism, and the glyoxylate shunt and Entner-Doudoroff pathway are active in the absence of *pgi* gene [9-11]. As expected, our results indicate that the flux for phosphoglucose isomerase (*r*_1_*r*_2_) decreased and those for the oxidative pentose phosphate pathway (*r*_24_) and Entner-Doudoroff pathway (*r*_33_) increased as the *pgi* expression levels decreased (Figures 4 and Additional file 2). The *r*_1_*r*_2_ values were negative, when *pgi* expression-conrollable strain YUEC04 with <20 μg/mL IPTG and the *pgi* knockout strain JWK3985 were cultured. However, since it is difficult to fit to near zero in the parameter fitting, and in these cases, the *r*_1_*r*_2_ values are considered to be approximately zero. Furthermore, the flux for phosphoglucose isomerase in YUEC04 strain cultured with IPTG was lower than that in the control strain YUEC00, but the *pgi* expression levels in the YUEC04 strain cultured with IPTG were higher than that in the YUEC00. The lower specific growth rate of the YUEC04 strain cultured with IPTG compared to YUEC00 seems consistent with the result of ^13^ C-MFA results. These phenomena might result from the different translational efficiencies of Pgi protein in the YUEC04 and YUEC00 strains.

In the anaplerotic reactions, net flux from phosphoenolpyruvate to oxaloacetate (*r*_19_–*r*_20_) gradually decreased as the *pgi* expression level decreased, but it was higher in the *pgi*-knockout strain JWK3985 than that in the *pgi* expression-controllable strain YUEC04 cultured without IPTG. Comparatively, the flux for the glyoxylate shunt (*r*_22_) increased as the *pgi* expression levels decreased, but it was lower in the *pgi*-knockout strain JWK3985 than that in the *pgi* expression-controllable strain YUEC04 cultured without IPTG. These phenomena were consistent with the expected expression levels of *aceBA* genes (Additional file 3). These results indicate that the flux for the glyoxylate shunt increases as the flux for phosphoglucose isomerase decreases due to decreased *pgi* expression levels; however the glyoxylate shunt flux decreases after adapting to the complete loss of *pgi* expression. It is known that both the phosphoenolpyruvate carboxylase reaction and the glyoxylate shunt are utilized for the supply of oxaloacetate to the TCA cycle; the phosphoenolpyruvate carboxylase reaction catalyze the conversion from phosphoenolpyruvate and CO2 to oxaloacetate, and the glyoxylate shunt also contribute to supplying oxaloacetate via glyoxylate, succinate, fumarate and malate by using isocitrate in the TCA cycle and acetyl-CoA which is produced by acetate catabolism. Our experimental results using the *pgi* expression-controllable strain suggest that E. coli might use the glyoxylate shunt rather than the phosphoenolpyruvate carboxylase reaction for maintaining intracellular oxaloacetate levels to adapt to the *pgi* gene knockout. No acetate production in *pgi* expression-controllable and *pgi* knockout strains also support this phenomenon (Table 1). Moreover, after adapting to the absence of phosphoglucose isomerase, both the phosphoenolpyruvate carboxylase reaction and the glyoxylate shunt are used to maintain intracellular oxaloacetate levels. These phenomena were consistent with the results of MSA, indicating that the perturbed expression of *pgi* gene has a large impact to the metabolic fluxes for the branch point between glycolysis and the oxidative pentose phosphate pathway (*r*_1_–*r*_2_ and *r*_23_) and for the anaplerotic reactions (*r*_19_–*r*_20_ and *r*_21_) (Table 3).

In addition, MSA revealed that the change in *pgi* expression has an impact to the metabolic flux of isocitrate dehydrogenase reaction (*r*_15_) (Table 3). This enzyme locates in the branch point between the TCA cycle and the glyoxylate shunt. It is known that the major metabolic pathways for NADPH production are NADP-dependent isocitrate dehydrogenase reaction and the oxidative pentose phosphate pathway in the wild-type *E. coli*. This isocitrate dehydrogenase reaction is inhibited by high NADPH concentration [18]. When the *pgi* expression was decreased, the metabolic flux toward the oxidative pentose phosphate pathway was increased. Therefore, it can be thought that NADP is highly produced via this pathway by decreased *pgi* expression (Figure 3 and Additional file 2). This result indicates that the decreased *pgi* expression leads to the decreased flux for the isocitrate dehydrogenase reaction due to high NADPH concentration produced via the oxidative pentose phosphate pathway, and main pathway for producing NADPH is shifted to the oxidative pentose pathway alone by decreased *pgi* expression. Although it is also known that malic enzyme reaction (*r*_21_) contributes to NADPH production [19], clear relationship between *pgi* expression levels and flux changes could not be observed.

In addition, it is known that *aceK* gene is located in the downstream of *aceBA* genes, and *aceBaceA* and *aceK* genes constitute an *aceBAK* operon in *E. coli* genome [20,21]. The *aceK* gene encodes isocitrate dehydrogenase kinase/phosphatase and involves in the inhibition of isocitrate dehydrogenase activity via phosphorylation. Therefore, it can be speculated that the expression of *aceK* gene as well as *aceBA* genes (Additional file 3) is increased by the decrease in *pgi* expression level and increased *aceBAK* operon expression is also related to the decrease in the metabolic flux for isocitrate dehydrogenase reaction and the increase in the flux for glyoxylate shunt.

We utilized our gene expression control system to analyze the effect of perturbations to the expression levels of the essential *E. coli* gene *eno* on metabolic flux. The *eno* gene encodes enolase, which catalyzes the conversion from 2-phosphoglycerate to phosphoenolpyruvate, and its knockout is lethal when grown on glucose as a carbon source. We successfully analyzed the effect of increased *eno* expression on cellular metabolism, but we could not determine the IPTG concentration, which achieved lower expression levels of *eno* gene than that measured in the control strain (data not shown). These results confirmed that maintenance of *eno* expression levels is important for the growth of *E. coli*. As shown in Figure 6 and Additional file 4, the metabolic flux distribution did not change when the *eno* expression level was increased, but the specific growth and glucose uptake rates slightly decreased. Our results indicate that overall activity of central metabolism is slightly decreased by increased *eno* expression levels, but the magnitude of the change in metabolic fluxes in E. coli is small due to the change in *eno* expression levels. This phenomenon was supported by the result of MSA; i.e. the e-FSCs for major metabolic reactions were nearly equal to zero except for the glyoxylate shunt (*r*_22_) and the Entner-Doudroff pathway (*r*_33_) (Table 4); the perturbed *eno* expression had a small impact to the flux for Entner-Doudoroff pathway. However, the reason of this phenomenon is obscure. In case of the glyoxylate shunt, since the absolute flux values were small in wild-type and *eno* expression-controllable strain, e-FSC for this reaction became large and this phenomenon is worthy of little attention.

Fong et al. completed ^13^ C-MFA on adaptively evolved *pgi*-knockout mutants, and suggested that the *pgi*-knockout strain can evolve to improved phenotypes such as increased glucose uptake rate and growth rate [22]. Moreover, genomic studies of adaptively evolved *pgi*-knockout mutant were also performed by Charusanti et al., indicating that frequent mutations in genomic DNA were found in the *pntAB* and *udhA* genes encoding membrane-bound and soluble transhydrogenases, respectively, which contribute to NADPH formation from NADH [23]. Our experimental system can be used to understand the transition from the initial state, to an altered state due to gene expression perturbations and to the state induced by the absence of an entire gene, and encourages genomic and metabolic analyses of the adaptation process to gene expression perturbations and supports the use of metabolic engineering for breeding useful microorganisms. This experimental system allows the active and inactive genes and/or metabolic reactions to be identified during adaptation process in response to perturbations and aids in the designing of desired microorganisms by metabolic engineering. It is expected that cell behaviors might be drastically altered by applying perturbations to the identified active and inactive genes and/or metabolic reactions, and therefore, the desired microorganisms can be obtained among such perturbed strains showing various phenotypic behaviors.

## Conclusions

We successfully analyzed the response of the central carbon metabolism network to the perturbations to the *pgi* and *eno* expression levels with our gene expression-controllable strains of *E. coli*. Our simple gene expression control system allows the initial cellular state to be determined and the initial cellular state for all examined experiments to be unified. Moreover, transition from the initial cellular state to the state induced following adaptation to altered target gene expression can be analyzed. Further analyses are required to understand the behavior of the metabolic network against gene expression perturbations.

This technique is amenable to stepwise increases and decreases in target gene expression levels and analysis of their effects on the metabolic network. Further to the work we have presented here, additional research regarding the response of other layers of the intracellular biological networks consisting of mRNA and protein expression, and metabolite synthesis as well as metabolic flux, to gene expression perturbations will lead to increased understanding of intracellular systems and more effective microorganism-breeding techniques for the production of useful chemicals.

## Methods

### Strains, media and culture condition

The *E. coli* BW25113 strain [Δ(*araD-araB*)*567* Δ*lacZ4787*(*::rrnB-3*) λ^–^*rph-1* Δ(*rhaD-rhaB*)*568 hsdR514* was used as a host for the construction of gene expression-controllable strains. To obtain P1kc phages for disrupting *pgi* gene, the *pgi*-knockout strain JWK3985 in the Keio collection [16] was used. For the recombinant DNA technique, *E. coli* DH5α [F^–^ Φ80d *lacZ*Δ*M15* Δ(*lacZYA-argF*)*U169 deoRrecA1 endA1hsdR17*(r_K_^–^, m_K_^+^) *phoA supE44* λ^–^*thi-1 gyrA96 relA1* was used.

For the construction of the gene expression-controllable strains, Lennox medium (1% Bactotryptone, 1% Bacto yeast extract, 0.5% NaCl, and 0.1% glucose, pH 7.2), SOB medium (2% Bacto tryptone, 0.5% Bacto yeast extract, 0.05% NaCl, 2.5 mM KCl, and 10 mM MgCl_2_) and SOC medium (SOB medium supplemented with 20 mM glucose) were used. When preparing the agar plate, 1.5% agar was added to the medium.

For analyzing the effect of gene expression perturbation on cellular metabolism, the *E. coli* strains were cultivated at 37°C in modified M9 medium (17.1 g/L Na_2_HPO_4_·12H_2_O, 3 g/L KH_2_PO_4_, 0.5 g/L NaCl, 2 g/L NH_4_Cl, 0.493 g/L MgSO_4_·7H_2_O, 4 g/L glucose, 10 mg/L thiamin·HCl, 2.78 mg/L FeSO_4_·7H_2_O, 14.7 mg/L CaCl_2_·2H_2_O) [24] in shaking flasks; a mixture of [U-^13^ C]glucose and [1-^13^ C]glucose at a ratio of 1:1 was used.

If necessary, 50 μg/mL ampicillin, 20 μg/mL kanamycin, 20 μg/mL chloramphenicol, and 1 mM L-arabinose were added to the culture medium.

### Construction of *pgi* and *eno* expression-controllable *E. Coli* strains

The DNA fragments encompassing the *pgi* and *eno* open reading frames were amplified by PCR using *Ex Taq* DNA polymerase (Takara Bio Inc., Shiga, Japan) or KOD -Plus- DNA polymerase (Toyobo Co., Ltd., Osaka, Japan) and sets of primers, 5'-GGCCCTGAGGGCCATTAAGAAAATCGGTGTGTTGAC-3' and 5'-GGCCGCATAGGCCAATACAGTTTTTTCGCGCAGTCC-3' for *pgi* and 5'-GGCCCTGAGGGCCTCCAAAATCGTAAAAATCATCGG-3' and 5'-GGCCGCATAGGCCTGCCTGGCCTTTGATCTCTTTACG-3' for *eno*. The fragments were cloned into the pGEM-T easy vector (Promega Co., Madison, WI), and the resulting plasmids were named pGEM-T-*pgi* and pGEM-T-*eno*. After confirming the sequence of the cloned DNA fragment using ABI Prism 3130 genetic analyzer (Applied Biosystems, Inc., Foster City, CA) and BigDye terminator cycle sequencing kit ver. 3.1 (Applied Biosystems), each gene fragment was excised by *Sfi*I digestion, and then cloned into the *Sfi*I sites of the mini-F plasmid pFE604. The resulting plasmids, named pFE604-*pgi* and pFE604-*eno*, respectively, were introduced into the *E. coli* BW25113 strain. After cultivating the transformant in the presence of 20 μg/mL IPTG to express the cloned gene, the target gene was disrupted in the presence of 20 μg/mL IPTG. The *pgi* gene was disrupted by P1kc phage-mediated transduction using the *pgi* knockout strain JWK3985. The *eno* gene was disrupted by the one-step inactivation method reported by Baba et al. [16] was adopted. Briefly, the kanamycin resistance gene with a homologous sequence to upstream and downstream regions of the *eno* open reading frame was amplified by PCR using the pKD13 plasmid as a template and a set of primers, 5'-TGGCATTTTTAAATCAGATAAAGTCAGTCTTATGCCTGGCCTTTGATCTCATTCCGGGGATCCGTCGACC-3' and 5'- TTGTTTGTCTGGAGTTTCAGTTTAACTAGTGACTTGAGGAAAACCTAATGTGTAGGCTGGAGCTGCTTCG-3'. Then, the amplified fragment was introduced into the BW25113/pKD46 carrying pFE604-*eno* cultured in SOC medium containing 1 mM arabinose and 20 μg/mL IPTG by electroporation, and kanamycin-resistant transformants were obtained. After disrupting the target gene, the pKD46 plasmid was cured from the above transformants, and the obtained strains were cultivated in the presence of 20 μg/mL IPTG and then stored at −80°C. The *pgi* and *eno* expression-controllable strains were named YUEC04 and YUEC01, respectively. The BW25113 transformed with pFE604 (BW25113/pFE604) was also constructed as a control strain, named YUEC00.

### Gene expression analysis by real-time RT-PCR

Gene expression analysis was completed on the total RNA isolated from exponentially growing *E. coli* cells using the RNeasy mini kit (Qiagen K.K., Tokyo, Japan), according to the manufacturer’s protocol. Reverse transcription was then performed on the isolated total RNA by using the PrimeScript RT reagent kit (Takara Bio Inc.). After appropriate dilution of the reverse transcription reaction mixture, real-time PCR was conducted using the StepOne Plus real time PCR system (Applied Biosystems) and the Fast SYBR green master mix (Applied Biosystems). Analysis was performed in triplicate using 2 independently synthesized cDNAs for each sample. For quantification, the standard DNA (linear plasmid DNA) was simultaneously amplified. Since absolute expression level of target gene for perturbation might change during cultivation, gene expression level of the target gene was determined as a ratio to that of the housekeeping gene *gapA*, expecting that this ratio keeps constant during growth phase. The primer sequences for real-time PCR were as follows: 5' CTGGCAGGCACTACAGAAACAC-3' and 5'-TCGTCGAAGGTTGCGGAGAAC-3' for *pgi*; 5'-GCTCCGTCAGGTGCTTCTAC-3' and 5'-CAGCAACAGCTTTGGTTACG-3' for *eno*; 5'-AGCGGGTATTGAAGCAGTCT-3' and 5'-GTTTGCCGGATAGAGCGACT-3' for *aceA*; 5'-TTCTGACTGAGCTGGTGACG-3' and 5'-TCAGGCAACGTTCCGTTATC-3' for *aceB*; 5'-TGAACGTGATCCGGCTAACCTG-3' and 5'-GCGGTGATGTGTTTACGAGCAG-3' for *gapA*.

The linear standard DNA was prepared using pGEM-T-*pgi* and pGEM-T-*eno* to quantify the *pgi* and *eno* gene expression levels. To construct the standard plasmid to quantify *aceA* and *aceB* expression levels, the DNA fragment encompassing the *aceA* or *aceB* genes was amplified by PCR using primers 5'-GGCCCTGAGGGCCAAAACCCGTACACAACAAATTG-3' and 5'-GGCCGCATAGGCCGAACTGCGATTCTTCAGTGGAG-3' for *aceA* and 5'-GGCCCTGAGGGCCACTGAACAGGCAACAACAACCG-3' and 5'- GGCCGCATAGGCCCGCTAACAGGCGGTAGCCTGGC -3' for *aceB*, and then cloned onto pGET-T easy plasmid. In the construction of the *gapA* standard plasmid, the DNA fragment containing *gapA* was amplified by PCR using primers 5'-GGCCCTGAGGGCCACTATCAAAGTAGGTATCAACGG-3' and 5'-GGCCGCATAGGCCTTTGGAGATGTGAGCGATCAGGTC-3', and then cloned onto pGEM-T easy plasmid. All the plasmids were digested with the *Nde*I restriction enzyme and purified the by MinElute reaction cleanup kit (Qiagen) to prepare the linear standard DNA for real-time RT-PCR.

### Measurement of cells, glucose and acetate concentrations

The cell concentration in the culture was determined by the following equation,

(1) $$\text{Cell concentration }\left(\text{g dry cell}/\text{L}\right)={\text{OD}}_{660}\times 0.33\;\left(\text{g dry cell}/\text{L}/{\text{OD}}_{660}\right)$$

The OD_660_ was measured using UVmini-1240 spectrophotometer (Shimadzu Corporation, Kyoto, Japan). The glucose concentration was measured using the Bioanalyzer BF-5 (Oji Scientific Instruments, Hyogo, Japan). The acetate concentration was measured using a high performance liquid chromatography (HPLC) system (Shimadzu Corporation) equipped with TSKgel OApak-A and TSKgel OApak-P columns (Tosoh Corporation, Tokyo, Japan). During HPLC analysis, the column temperature was kept at 40°C, and the mobile phase, 0.75 mM H_2_SO_4_ solution, was run at 0.8 mL/min, and the eluent absorbance was detected at 210 nm. In this analysis, pyruvate, lactate, formate, succinate, fumarate and malate as well as acetate can be analyzed. However, only acetate was detected in culture supernatant (data not shown).

### Gas chromatograph/mass spectrometry analysis

*E. coli* cells were harvested during the exponential growth phase in the medium containing ^13^ C-labeled glucose by centrifugation and hydrolyzed in 6 M HCl at 105°C for 24 h. After removing the debris by filtration, the hydrolysate was dried, and the resulting material containing amino acids was dissolved in acetonitrile. For derivatization of the proteinogenic amino acids, the hydrolasate was mixed with an equal volume of *N-*(*tert*-butyldimethylsilyl)-*N*-methyl-trifluoroacetamide, and then the mixture was incubated at 100°C for 1 h.

For gas chromatograph/mass spectrometry (GC/MS) analysis, 7890A GC and 5975C MSD systems (Agilent Technologies, Santa Clara, CA) equipped with DB-5MS-DG column (30-m length, 0.25-mm inside diameter, and 0.25-μm thickness; Agilent Technologies) was used. Helium gas was used as a carrier at a flow rate of 1 mL/min. The interface and ion source temperatures were set at 250°C and 200°C, respectively. The electron impact voltage was set to 70 eV. The column temperature was initially set at 150°C for 2 min, and then increased to 280°C at the rate of 3°C/min. In this study, four fragment ions, namely, [M–57]^+^, [M–85]^+^, [M–159]^+^, and [f302]^+^, of *tert*-butyldimethylsilylated amino acids were monitored in the selected ion monitoring mode. The GC/MS data were corrected considering the natural abundance of C, H, N, O, and Si isotopes for metabolic flux calculations [25].

### ^13^ C-metabolic flux analysis

The metabolic flux was determined using a metabolic reaction model, including glycolysis, pentose phosphate pathway, TCA cycle, anaplerotic reactions, biosynthesis pathways of organic acids such as lactate, acetate, formate and succinate, ethanol biosynthesis pathway and the Entner-Doudoroff pathway was constructed (Figure 3 and Additional file 5). *E. coli* can produce acetate from pyruvate by PoxB protein as well as from acetyl-CoA by Pta and AckA proteins[26-29]. However, in ^13^C-MFA, the fluxes to acetate from pyruvate or acetyl-CoA cannot be resolved to determine; i.e. the ^13^C-labeling information in acetate from acetyl-CoA is the same as that from pyruvate. Therefore, we considered the acetate biosynthesis pathway from acetyl-CoA only.

The metabolic flux distribution was determined as follows. Assuming the metabolic fluxes is in a steady state, 13 independent fluxes (*r*_2_, *r*_5_, *r*_7_, *r*_9_, *r*_11_, *r*_18_, *r*_20_, *r*_21_, *r*_23_, *r*_28_, *r*_30_, *r*_32_, and *r*_33_) were iteratively tuned by minimizing the following residual *R*,

(2) $$R={\sum_{i}\left(\frac{M_{i}-M_{i\text{, simulated}}}{M_{i}}\right)}^{2}$$

where *M*_*i*_ and *M*_*i*, simulated_ represent the measured and simulated GC/MS data of the *i*-th amino acid, respectively. In general, the residual R calculated by equation (2) has been weighted by experimental errors of GC/MS analysis. In our GC/MS measurement, however, experimental errors of GC/MS analysis of proteinogenic amino acids were similar to each other. Therefore, we did not use an error-weighted criterion for calculating the residual *R* by equation (2) to be minimized.

The metabolic flux distribution was determined from the calculated independent fluxes and the fluxes from metabolic intermediates to biomass synthesis (*r*_35_*r*_36_*r*_37_*r*_38_*r*_39_*r*_40_*r*_41_*r*_42_*r*_43_*r*_44_, and *r*_45_; Additional file 6) calculated by

(3) $$\text{Flux for biomass synthesis }=Y_{X}{}_{/S}\times W\times \;100$$

where *YX/S* represents the biomass yield (g dry cell produced/mmol glucose consumed) and *W* represent the anabolic demand for unit biomass synthesis (mmol metabolite/g dry cell) described in the literatures [30]. In this study, we used the same values of anabolic demand for biomass synthesis, and it was confirmed that the estimation errors did not show significant difference among the different gene experiments and strains (data not shown) and the flux distributions were correctly determined. All fluxes were normalized to the specific glucose uptake rate of 100, to allow comparison of the flux distributions at different glucose uptake rates.

For iterative calculation, the Levenberg-Marquart method was adopted. All calculations were conducted using Matlab 2010b (The Mathworks Inc., Natick, MA). In this study, metabolic flux distributions in 3 independent cultivations of each *E. coli* strain were determined.

### Metabolic sensitivity analysis

To verify the impact of perturbed gene expression on metabolic flux, MSA was performed. In this study, e-FSC was calculated by the following equation

(4) $$C_{i}^{J_{k}}=\frac{{\mathrm{\partial J}}_{k}/J_{k}}{{\mathrm{\partial G}}_{i}/G_{i}}=\frac{{\mathrm{\partial J}}_{k}}{{\mathrm{\partial G}}_{i}}\frac{G_{i}}{J_{k}}$$

where $C_{i}^{J_{k}}$ represents the e-FSC for reaction *r*_k_ (see Figure 3 and Additional file 5) when perturbing to the expression of gene *i* (*pgi* or *eno* in this study), and *J*_*k*_ and *G*_*i*_ represent the flux for reaction *r*_*k*_, and the expression level of gene *i*, respectively. Similarly, in case of estimation of the e-FSC for the net flux of *r*_*k*_ and its reversed reaction *r*_*k*+1_*J*_*k*_*J*_*k*+1_, which represents the net flux for *r*_*k*_ and *r*_*k*+1_, is substituted into *J*_*k*_ in equation (4). For estimation of e-FSCs, a free software package R [31] and Microsoft Excel (Microsoft Corporation, Redmond, WA) were used.

## Competing interests

The authors declare that they have no competing interests.

## Authors’ contributions

YU carried out the strain construction, gene expression analysis and metabolic flux analysis. TH participated in the design of the study, performed metabolic sensitivity analysis, and drafted the manuscript. TS, CF, NY, and HM participated in the design of the study. HS conceived and supervised the study. All authors read and approved the final manuscript.

## Supplementary Material

Additional file 1 **Metabolic flux distribution of the*E. coli*YUEC00 (BW25113/pFE604) strain.** The values represent fluxes ± standard deviation from 3 independent culture experiments. All fluxes were normalized to the specific glucose uptake rate of 100. The production of ethanol, lactate and succinate are not shown because the fluxes for these reactions approximated to zero. G6P, glucose-6-phosphate; F6P, fructose-6-phosphate; FBP, fructose-1,6-bisphosphate; GAP, glyceraldehyde-3-phosphate, DHAP, dyhydroxyacetonephosphate; PGA, 3-phosphoglycerate; PEP, phosphoenolpyruvate; Pyr, pyruvate; AcCoA, acetyl-CoA; IsoCit, isocitrate; 2OG, 2-oxoglutarate; Suc, succinate; Mal, malate; Oxa, oxaloacetate; 6PG, 6-phosphogluconate; Ru5P, ribulose-5-phosphate; R5P, ribose-5-phosphate; S7P, sedohetulose-7-phosphate; X5P, xylulose-5-phosphate; E4P, erythrose-4-phosphate; KDPG, 2-keto-3-deoxyphosphogluconate. Click here for file

Additional file 2 **Metabolic flux distribution of the*pgi*expression-controllable strain YUEC04 and the*pgi*knockout strain JWK3985.** The values represent fluxes ± standard deviation from 3 independent culture experiments. All fluxes were normalized to specific glucose uptake rate of 100. The production of ethanol, lactate and succinate are not shown because the fluxes for these reactions approximated to zero. G6P, glucose-6-phosphate; F6P, fructose-6-phosphate; FBP, fructose-1,6-bisphosphate; GAP, glyceraldehyde-3-phosphate; DHAP, dyhydroxyacetonephosphate; PGA, 3-phosphoglycerate; PEP, phosphoenolpyruvate; Pyr, pyruvate; AcCoA, acetyl-CoA; IsoCit, isocitrate; 2OG, 2-oxoglutarate; Suc, succinate; Mal, malate; Oxa, oxaloacetate; 6PG, 6-phosphogluconate; Ru5P, ribulose-5-phosphate; R5P, ribose-5-phosphate; S7P, sedohetulose-7-phosphate; X5P, xylulose-5-phosphate; E4P, erythrose-4-phosphate; KDPG, 2-keto-3-deoxyphosphogluconate. Click here for file

Additional file 3 **Expression of*aceBA*genes in the*pgi*expression-controllable strain YUEC04, control strain YUEC00 and the*pgi*knockout strain JWK3985. Gene expression was analyzed using real time RT-PCR.** The expression levels of *aceB* (white bars) and *aceA* (solid bars) are shown. Click here for file

Additional file 4 **Metabolic flux distribution of the*eno*** expression-controllable strain YUEC01. The values represent fluxes ± standard deviation from 3 independent culture experiments. All fluxes were normalized by specific glucose uptake rate of 100. The production of ethanol, lactate and succinate are not shown because the fluxes for these reactions approximated to zero. G6P, glucose-6-phosphate; F6P, fructose-6-phosphate; FBP, fructose-1,6-bisphosphate; GAP, glyceraldehyde-3-phosphate; DHAP, dyhydroxyacetonephosphate; PGA, 3-phosphoglycerate; PEP, phosphoenolpyruvate; Pyr, pyruvate; AcCoA, acetyl-CoA; IsoCit, isocitrate; 2OG, 2-oxoglutarate; Suc, succinate; Mal, malate; Oxa, oxaloacetate; 6PG, 6-phosphogluconate; Ru5P, ribulose-5-phosphate; R5P, ribose-5-phosphate; S7P, sedohetulose-7-phosphate; X5P, xylulose-5-phosphate; E4P, erythrose-4-phosphate; KDPG, 2-keto-3-deoxyphosphogluconate. Click here for file

Additional file 5 **Metabolic reaction model of *E. coli*for**^**13**^** C-MFA.** Reaction names colored in red represent the independent flux in MFA. G6P, glucose-6-phosphate; F6P, fructose-6-phosphate; FBP, fructose-1,6-bisphosphate; GAP, glyceraldehyde-3-phosphate; DHAP, dyhydroxyacetonephosphate; PGA, 3-phosphoglycerate; PEP, phosphoenolpyruvate; Pyr, pyruvate; AcCoA, acetyl-CoA; IsoCit, isocitrate; 2OG, 2-oxoglutarate; Suc, succinate; Mal, malate; Oxa, oxaloacetate; 6PG, 6-phosphogluconate; Ru5P, ribulose-5-phosphate; R5P, ribose-5-phosphate; S7P, sedohetulose-7-phosphate; X5P, xylulose-5-phosphate; E4P, erythrose-4-phosphate; KDPG, 2-keto-3-deoxyphosphogluconate. Click here for file

Additional file 6 **Fluxes from metabolic intermediates to biomass synthesis in the control strain YUEC00,*pgi*expression-controllable strain YUEC04,*pgi*knockout strain JWK3985, and*eno*expression controllable strain YUEC01.** The fluxes from intermediate metabolites to biomass synthesis (*r*_35_, *r*_36_, *r*_37_, *r*_38_, *r*_39_, *r*_40_, *r*_41_, *r*_42_, *r*_43_, *r*_44_, and *r*_45_) in YUEC00 (A), YUEC04 and JWK3985 (B) and YUEC01 (C) strains are shown. The values represent fluxes ± standard deviation from 3 independent culture experiments. All fluxes were normalized to specific glucose uptake rate of 100. Click here for file
