# Supplementary figures and images for: Investigating the effects of perturbations to pgi and eno gene expression on central carbon metabolism in Escherichia coli using 13 C metabolic flux analysis
Source: Microb Cell Fact. 2012 Jun 21;11:87. doi: 10.1186/1475-2859-11-87 (PMC3778843; doi:10.1186/1475-2859-11-87)

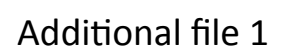

Supplement: Additional file 1 — Metabolic flux distribution of theE. coliYUEC00 (BW25113/pFE604) strain. The values represent fluxes ± standard deviation from 3 independent culture experiments. All fluxes were normalized to the specific glucose uptake rate of 100. The production of ethanol, lactate and succinate are not shown because the fluxes for these reactions approximated to zero. G6P, glucose-6-phosphate; F6P, fructose-6-phosphate; FBP, fructose-1,6-bisphosphate; GAP, glyceraldehyde-3-phosphate, DHAP, dyhydroxyacetonephosphate; PGA, 3-phosphoglycerate; PEP, phosphoenolpyruvate; Pyr, pyruvate; AcCoA, acetyl-CoA; IsoCit, isocitrate; 2OG, 2-oxoglutarate; Suc, succinate; Mal, malate; Oxa, oxaloacetate; 6PG, 6-phosphogluconate; Ru5P, ribulose-5-phosphate; R5P, ribose-5-phosphate; S7P, sedohetulose-7-phosphate; X5P, xylulose-5-phosphate; E4P, erythrose-4-phosphate; KDPG, 2-keto-3-deoxyphosphogluconate. [file 1475-2859-11-87-S1.pdf]

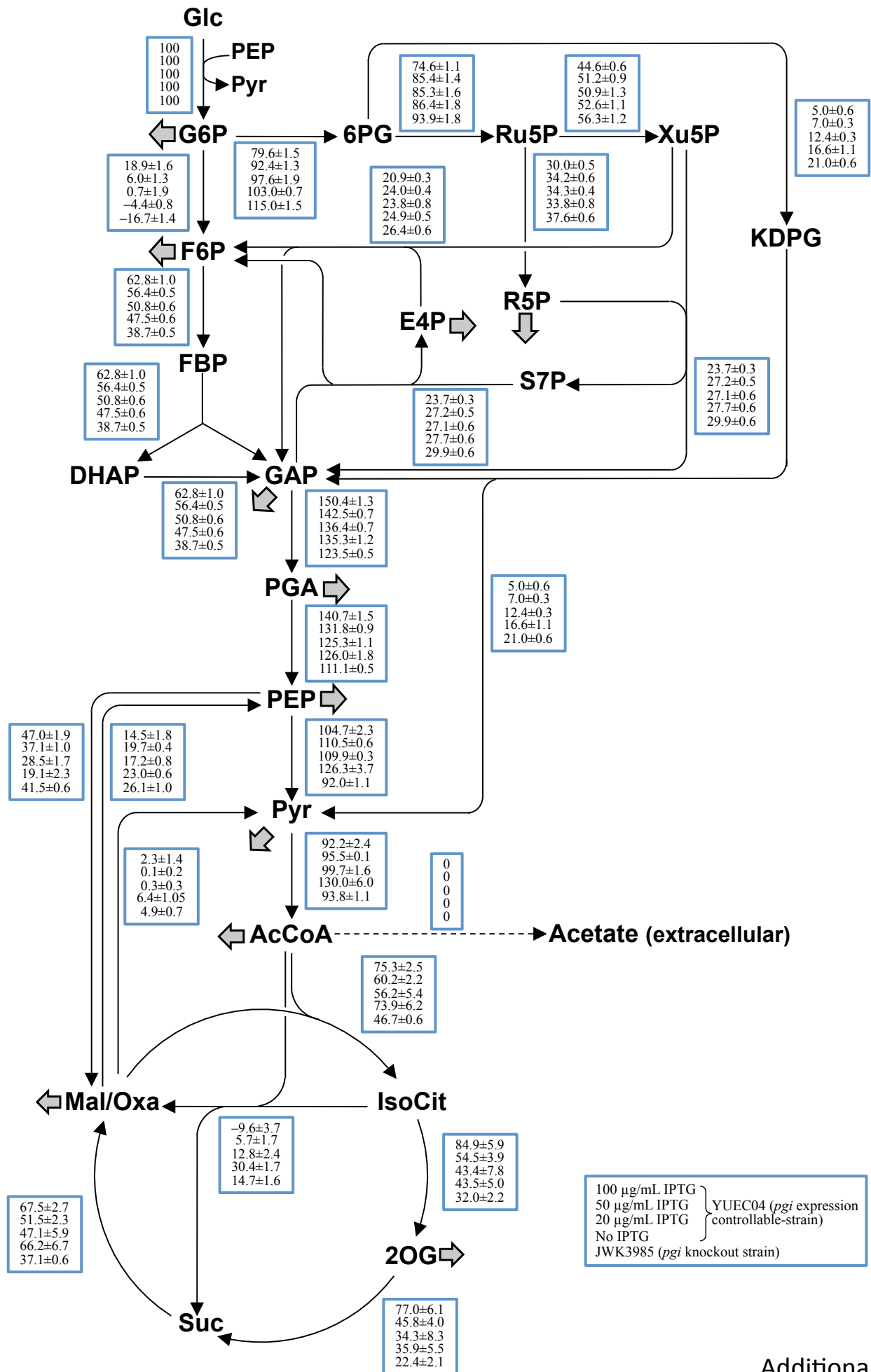

Supplement: Additional file 2 — Metabolic flux distribution of thepgiexpression-controllable strain YUEC04 and thepgiknockout strain JWK3985. The values represent fluxes ± standard deviation from 3 independent culture experiments. All fluxes were normalized to specific glucose uptake rate of 100. The production of ethanol, lactate and succinate are not shown because the fluxes for these reactions approximated to zero. G6P, glucose-6-phosphate; F6P, fructose-6-phosphate; FBP, fructose-1,6-bisphosphate; GAP, glyceraldehyde-3-phosphate; DHAP, dyhydroxyacetonephosphate; PGA, 3-phosphoglycerate; PEP, phosphoenolpyruvate; Pyr, pyruvate; AcCoA, acetyl-CoA; IsoCit, isocitrate; 2OG, 2-oxoglutarate; Suc, succinate; Mal, malate; Oxa, oxaloacetate; 6PG, 6-phosphogluconate; Ru5P, ribulose-5-phosphate; R5P, ribose-5-phosphate; S7P, sedohetulose-7-phosphate; X5P, xylulose-5-phosphate; E4P, erythrose-4-phosphate; KDPG, 2-keto-3-deoxyphosphogluconate. [file 1475-2859-11-87-S2.pdf]

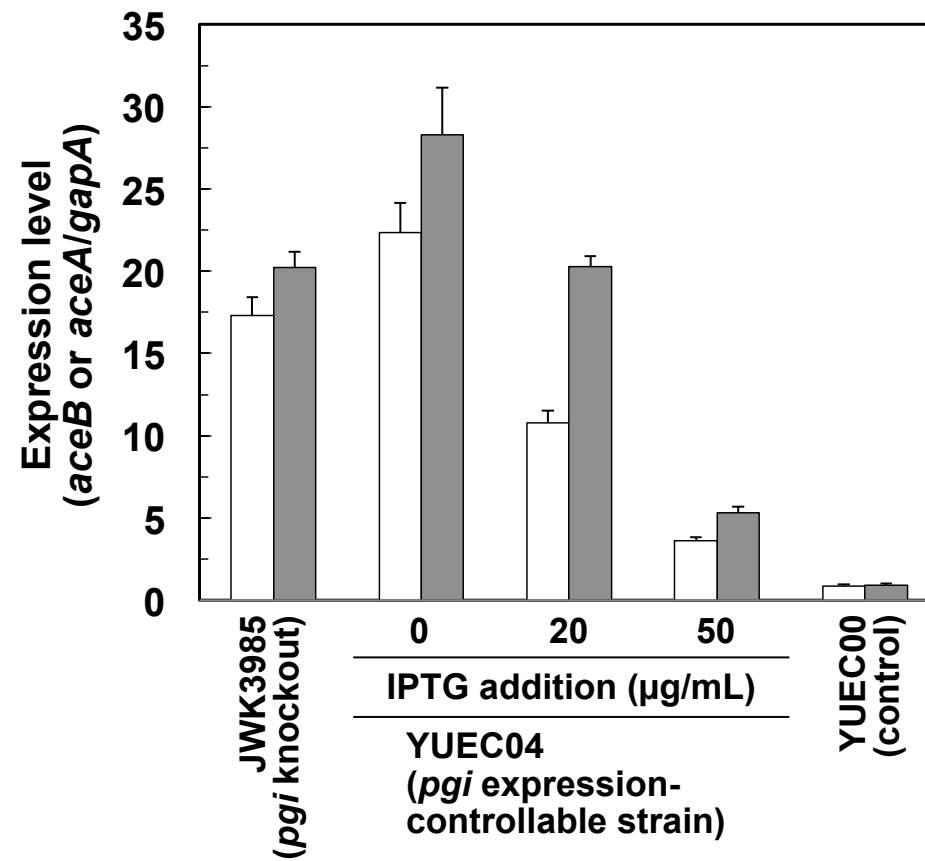

Supplement: Additional file 3 — Expression ofaceBAgenes in thepgiexpression-controllable strain YUEC04, control strain YUEC00 and thepgiknockout strain JWK3985. Gene expression was analyzed using real time RT-PCR. The expression levels of aceB (white bars) and aceA (solid bars) are shown. [file 1475-2859-11-87-S3.pdf]

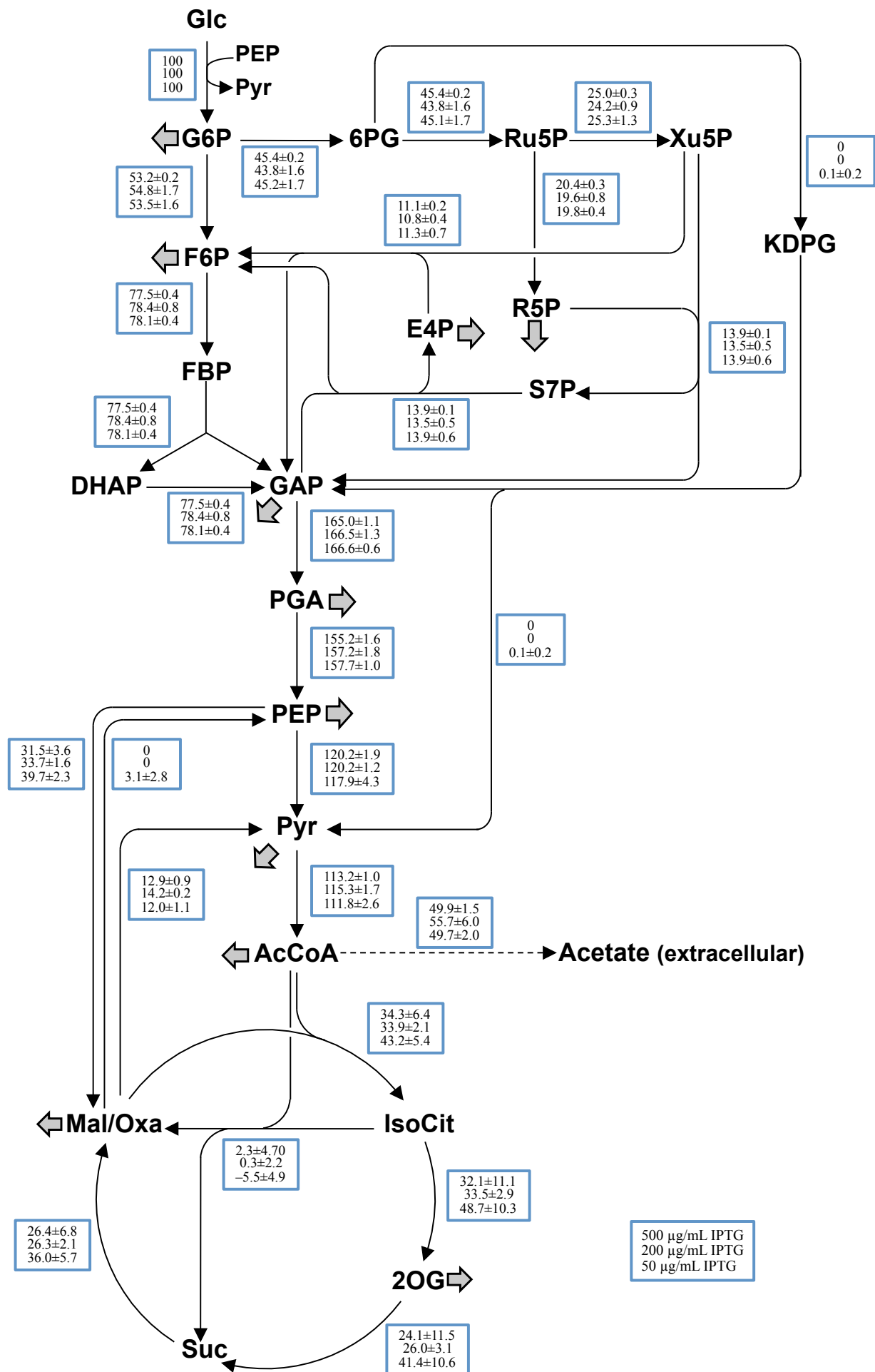

Supplement: Additional file 4 — Metabolic flux distribution of theeno expression-controllable strain YUEC01. The values represent fluxes ± standard deviation from 3 independent culture experiments. All fluxes were normalized by specific glucose uptake rate of 100. The production of ethanol, lactate and succinate are not shown because the fluxes for these reactions approximated to zero. G6P, glucose-6-phosphate; F6P, fructose-6-phosphate; FBP, fructose-1,6-bisphosphate; GAP, glyceraldehyde-3-phosphate; DHAP, dyhydroxyacetonephosphate; PGA, 3-phosphoglycerate; PEP, phosphoenolpyruvate; Pyr, pyruvate; AcCoA, acetyl-CoA; IsoCit, isocitrate; 2OG, 2-oxoglutarate; Suc, succinate; Mal, malate; Oxa, oxaloacetate; 6PG, 6-phosphogluconate; Ru5P, ribulose-5-phosphate; R5P, ribose-5-phosphate; S7P, sedohetulose-7-phosphate; X5P, xylulose-5-phosphate; E4P, erythrose-4-phosphate; KDPG, 2-keto-3-deoxyphosphogluconate. [file 1475-2859-11-87-S4.pdf]
