# Supplementary material for: Investigating the effects of perturbations to pgi and eno gene expression on central carbon metabolism in Escherichia coli using 13 C metabolic flux analysis
Source: Microb Cell Fact. 2012 Jun 21;11:87. doi: 10.1186/1475-2859-11-87 (PMC3778843; doi:10.1186/1475-2859-11-87)
Supplement: Additional file 5 — Metabolic reaction model of E. colifor13 C-MFA. Reaction names colored in red represent the independent flux in MFA. G6P, glucose-6-phosphate; F6P, fructose-6-phosphate; FBP, fructose-1,6-bisphosphate; GAP, glyceraldehyde-3-phosphate; DHAP, dyhydroxyacetonephosphate; PGA, 3-phosphoglycerate; PEP, phosphoenolpyruvate; Pyr, pyruvate; AcCoA, acetyl-CoA; IsoCit, isocitrate; 2OG, 2-oxoglutarate; Suc, succinate; Mal, malate; Oxa, oxaloacetate; 6PG, 6-phosphogluconate; Ru5P, ribulose-5-phosphate; R5P, ribose-5-phosphate; S7P, sedohetulose-7-phosphate; X5P, xylulose-5-phosphate; E4P, erythrose-4-phosphate; KDPG, 2-keto-3-deoxyphosphogluconate. [file 1475-2859-11-87-S5.pdf]

Additional file 5. Metabolic reaction model of *E. coli* for ^{13}C-MFA

Name	Reaction
Phosphotransferase system	
r_0	Glc + PEP => G6P + Pyr
Glycolysis	
r_1	G6P => F6P
r_2	F6P => G6P
r_3	F6P => FBP
r_4	FBP => GAP + DHAP
r_5	GAP + DHAP => FBP
r_6	DHAP => GAP
r_7	GAP => DHAP
r_8	GAP => PGA
r_9	PGA => GAP
r_{10}	PGA => PEP
r_{11}	PEP => PGA
r_{12}	PEP => Pyr
TCA cycle	
r_{13}	Pyr => AcCoA
r_{14}	AcCoA + Mal/Oxa => IsoCit
r_{15}	IsoCit => 2OG
r_{16}	2OG => Suc
r_{17}	Suc => Mal/Oxa
r_{18}	Mal/Oxa => Suc
Anaplerotic pathways and gluconeogenesis	
r_{19}	PEP + CO_2 => Mal/Oxa
r_{20}	Mal/Oxa => PEP + CO_2
r_{21}	Mal/Oxa => Pyr + CO_2
r_{22}	IsoCit + AcCoA => Suc + Mal/Oxa

Name	Reaction
Pentose phosphate pathway	
r_{23}	G6P => 6PG
r_{24}	6PG => Ru5P + CO_2
r_{25}	Ru5P => R5P
r_{26}	Ru5P => X5P
r_{27}	R5P + X5P => S7P + GAP
r_{28}	S7P + GAP => R5P + X5P
r_{29}	S7P + GAP => E4P + F6P
r_{30}	E4P + F6P => S7P + GAP
r_{31}	X5P + E4P => F6P + GAP
r_{32}	F6P + GAP => X5P + E4P
Entner-Doudoroff pathway	
r_{33}	6PG => KDPG
r_{34}	KDPG => GAP + Pyr
Biomass and organic acid synthesis	
r_{35}	G6P => Biomass
r_{36}	F6P => Biomass
r_{37}	GAP => Biomass
r_{38}	PGA => Biomass
r_{39}	PEP => Biomass
r_{40}	Pyr => Biomass
r_{41}	AcCoA => Biomass
r_{42}	2OG => Biomass
r_{43}	Mal/Oxa => Biomass
r_{44}	R5P => Biomass
r_{45}	E4P => Biomass
r_{46}	Pyr => Lactate (extracellular)
r_{47}	Pyr => Formate (extracellular) + AcCoA
r_{48}	AcCoA => Acetate (extracellular)
r_{49}	AcCoA => Ethanol (extracellular)
r_{50}	Suc => Succinate (extracellular)

Reaction names colored in red represent the independent flux in MFA. G6P, glucose-6-phosphate; F6P, fructose-6-phosphate; FBP, fructose-1,6-bisphosphate; GAP, glyceraldehyde-3-phosphate; DHAP, dyhydroxyacetonephosphate; PGA, 3-phosphoglycerate; PEP, phosphoenolpyruvate; Pyr, pyruvate; AcCoA, acetyl-CoA; IsoCit, isocitrate; 2OG, 2-oxoglutarate; Suc, succinate; Mal, malate; Oxa, oxaloacetate; 6PG, 6-phosphogluconate; Ru5P, ribulose-5-phosphate; R5P, ribose-5-phosphate; S7P, sedohetulose-7-phosphate; X5P, xylulose-5-phosphate; E4P, erythrose-4-phosphate; KDPG, 2-keto-3-deoxyphosphogluconate.
