# Supplementary material for: Investigating the effects of perturbations to pgi and eno gene expression on central carbon metabolism in Escherichia coli using 13 C metabolic flux analysis
Source: Microb Cell Fact. 2012 Jun 21;11:87. doi: 10.1186/1475-2859-11-87 (PMC3778843; doi:10.1186/1475-2859-11-87)
Supplement: Additional file 6 — Fluxes from metabolic intermediates to biomass synthesis in the control strain YUEC00,pgiexpression-controllable strain YUEC04,pgiknockout strain JWK3985, andenoexpression controllable strain YUEC01. The fluxes from intermediate metabolites to biomass synthesis (r35, r36, r37, r38, r39, r40, r41, r42, r43, r44, and r45) in YUEC00 (A), YUEC04 and JWK3985 (B) and YUEC01 (C) strains are shown. The values represent fluxes ± standard deviation from 3 independent culture experiments. All fluxes were normalized to specific glucose uptake rate of 100. [file 1475-2859-11-87-S6.pdf]

Additional file 6. Fluxes from metabolic intermediates to biomass synthesis in the control strain YUEC00, *pgi* expression-controllable strain YUEC04, *pgi* knockout strain JWK3985, and *eno* expression controllable strain YUEC01.

(A) Control strain (YUEC00)

Name	Reaction	Flux[a]
r_{35}	G6P => Biomass	1.4 ± 0.0
r_{36}	F6P => Biomass	0.7 ± 0.0
r_{37}	GAP => Biomass	1.1 ± 0.0
r_{38}	PGA => Biomass	9.4 ± 0.2
r_{39}	PEP => Biomass	3.4 ± 0.0
r_{40}	Pyr => Biomass	19.2 ± 0.4
r_{41}	AcCoA => Biomass	25.6 ± 0.5
r_{42}	2OG => Biomass	7.6 ± 0.2
r_{43}	Mal/Oxa => Biomass	12.4 ± 0.3
r_{44}	R5P => Biomass	6.2 ± 0.1
r_{45}	E4P => Biomass	2.7 ± 0.1

(B) *pgi* knockout (JWK3985) and *pgi* expression-controllable (YUEC04) strains

Name	Reaction	Flux[a]				
		JWK3985	YUEC04			
			0 μg/mL[b]	20 μg/mL[b]	50 μg/mL[b]	100 μg/mL[b]
r_{35}	G6P => Biomass	1.7 ± 0.0	1.4 ± 0.1	1.6 ± 0.1	1.6 ± 0.0	1.4 ± 0.1
r_{36}	F6P => Biomass	0.9 ± 0.0	0.7 ± 0.0	0.8 ± 0.1	0.8 ± 0.0	0.7 ± 0.0
r_{37}	GAP => Biomass	1.3 ± 0.0	1.1 ± 0.1	1.2 ± 0.1	1.2 ± 0.0	1.1 ± 0.0
r_{38}	PGA => Biomass	11.8 ± 0.0	9.4 ± 0.6	11.1 ± 0.6	10.7 ± 0.0	9.7 ± 0.2
r_{39}	PEP => Biomass	4.3 ± 0.0	3.4 ± 0.2	4.1 ± 0.2	3.9 ± 0.1	3.5 ± 0.1
r_{40}	Pyr => Biomass	24.2 ± 0.0	19.3 ± 1.2	22.9 ± 1.2	22.1 ± 0.4	19.8 ± 0.5
r_{41}	AcCoA => Biomass	32.4 ± 0.0	25.8 ± 1.6	30.6 ± 1.6	29.5 ± 0.5	26.5 ± 0.6
r_{42}	2OG => Biomass	9.6 ± 0.0	7.6 ± 0.5	9.1 ± 0.5	8.7 ± 0.2	7.8 ± 0.2
r_{43}	Mal/Oxa => Biomass	15.7 ± 0.0	12.5 ± 0.7	14.8 ± 0.8	14.3 ± 0.2	12.8 ± 0.3
r_{44}	R5P => Biomass	7.8 ± 0.0	6.2 ± 0.4	7.4 ± 0.4	7.1 ± 0.1	6.3 ± 0.2
r_{45}	E4P => Biomass	3.5 ± 0.0	2.8 ± 0.2	3.3 ± 0.2	3.2 ± 0.1	2.8 ± 0.1

(C) *eno* expression-controllable strain (YUEC01)

Name	Reaction	Flux[a]		
		50 μg/mL[b]	200 μg/mL[b]	500 μg/mL[b]
r_{35}	G6P => Biomass	1.3 ± 0.1	1.4 ± 0.1	1.4 ± 0.1
r_{36}	F6P => Biomass	0.7 ± 0.1	0.7 ± 0.1	0.7 ± 0.0
r_{37}	GAP => Biomass	1.0 ± 0.1	1.1 ± 0.1	1.1 ± 0.1
r_{38}	PGA => Biomass	8.9 ± 0.4	9.3 ± 0.4	9.7 ± 0.6
r_{39}	PEP => Biomass	3.2 ± 0.1	3.4 ± 0.4	3.5 ± 0.2
r_{40}	Pyr => Biomass	18.3 ± 0.9	19.0 ± 0.9	19.9 ± 1.1
r_{41}	AcCoA => Biomass	24.4 ± 1.1	25.4 ± 1.1	26.7 ± 1.4
r_{42}	2OG => Biomass	7.2 ± 0.3	7.5 ± 0.3	7.9 ± 0.4
r_{43}	Mal/Oxa => Biomass	11.8 ± 0.5	12.3 ± 0.6	12.9 ± 0.7
r_{44}	R5P => Biomass	5.9 ± 0.3	6.1 ± 0.3	6.4 ± 0.3
r_{45}	E4P => Biomass	2.6 ± 0.1	2.7 ± 0.1	2.9 ± 0.2

[a]Flux value from metabolic intermediates to biomass formation normalized by specific glucose uptake rate as 100 is shown.
[b]IPTG concentration added to culture is shown.
